# Can Biomarker Assessment on Circulating Tumor Cells Help Direct Therapy in Metastatic Breast Cancer?

**DOI:** 10.3390/cancers6020684

**Published:** 2014-03-25

**Authors:** Natalie Turner, Marta Pestrin, Francesca Galardi, Francesca De Luca, Luca Malorni, Angelo Di Leo

**Affiliations:** 1 Sandro Pitigliani Medical Oncology Department, Prato Hospital, Istituto Toscano Tumori, Via Ugo Foscolo, Prato, PO 59100, Italy; E-Mails: nhturner@usl4.toscana.it (N.T.); mpestrin@usl4.toscana.it (M.P.); lmalorni@usl4.toscana.it (L.M.); 2 Translational Research Laboratory, Prato Hospital, Via Ugo Foscolo, Prato, PO 59100, Italy; E-Mails: fgalardi@usl4.toscana.it (F.G.); frdeluca@usl4.toscana.it (F.L.)

**Keywords:** biomarker, breast cancer, CellSearch, chemotherapy, circulating tumor cells, HER2, liquid biopsy, metastatic, predictive biomarker, anti-HER2 therapy

## Abstract

Circulating tumor cell (CTC) count has prognostic significance in metastatic breast cancer, but the predictive utility of CTCs is uncertain. Molecular studies on CTCs have often been limited by a low number of CTCs isolated from a high background of leukocytes. Improved enrichment techniques are now allowing molecular characterisation of single CTCs, whereby molecular markers on single CTCs may provide a real-time assessment of tumor biomarker status from a blood test or “liquid biopsy”, potentially negating the need for a more invasive tissue biopsy. The predictive ability of CTC biomarker analysis has predominantly been assessed in relation to HER2, with variable and inconclusive results. Limited data exist for other biomarkers, such as the estrogen receptor. In addition to the need to define and validate the most accurate and reproducible method for CTC molecular analysis, the clinical relevance of biomarkers, including gain of HER2 on CTC after HER2 negative primary breast cancer, remains uncertain. This review summarises the currently available data relating to biomarker evaluation on CTCs and its role in directing management in metastatic breast cancer, discusses limitations, and outlines measures that may enable future development of this approach.

## 1. Introduction

Breast cancer incidence in Western countries is estimated at around 125 per 100,000 women, of which 5% present with *de novo* metastatic disease [1,2], while a significant minority of women with early breast cancer develop recurrence after adjuvant or neoadjuvant systemic therapy [3]. Despite improvements in treatment options, breast cancer remains one of the leading causes of cancer mortality in women, with 5-year mortality from metastatic breast cancer (MBC) estimated at less than 25% [1]. With more than 90% of cancer mortality due to development of metastatic disease rather than due to the primary cancer itself [4], continued development and improvement of MBC treatments are critical. A field of growing interest is that of circulating tumor cells (CTCs), which may provide useful prognostic and predictive information to guide treatment decisions.

CTCs are tumor cells that have escaped from the primary (or metastatic) tumor into the blood. As this is a critical step in the ability for cancers to metastasise, CTCs are considered potential precursors of metastatic disease [4] or “metastatic intermediaries” [5]. CTCs were first described over 140 years ago by Ashworth [6], yet it has not been until relatively recently that technologies for reliable identification and isolation of CTCs have been developed, leading to a significant increase in interest in their potential clinical utility.

The prognostic role of CTCs in MBC is now well established. In a landmark study from Cristofanilli *et al*. [7], women with MBC who had a CTC count of ≥5 per 7.5 mL of whole blood had significantly shorter progression free (PFS) and overall survival (OS) compared with women with CTC count of <5, both prior to start of treatment and after repeat assessment at four weeks. Additional studies in MBC using this same cut-off to define high CTC count have reported consistent findings [8,9,10]. Furthermore, CTCs, rather than disseminated tumor cells (*i.e*., isolated tumor cells detectable in bone marrow), appear to be a better prognostic marker in advanced breast cancer [11]. While data are less robust in early breast cancer, evidence to date also supports an association between high CTC count and poorer outcomes [12,13]. 

Although CTCs can provide prognostic information in breast cancer, their utility as predictive markers is less certain. CTC count has been suggested as a means of monitoring therapeutic response to treatment. Patients with an elevated CTC count prior to commencing therapy, which subsequently does not decrease following treatment, have been shown to have poorer outcomes, which might therefore predict treatment resistance [14,15]. In a study of nearly 100 patients with MBC by Martin *et* *al*., CTC count after the 1st cycle of chemotherapy was the only independent factor significantly associated with OS or PFS [15]. Similarly, lack of CTC response, even in the setting of a radiological response, portends a worse prognosis [16]. Based on this concept, the predictive role of CTC count is currently being investigated in the SWOG 0500 randomised phase III trial (*NCT00382018*), which aims to enrol 651 patients with MBC, and is addressing the question of whether or not a treatment regimen should be altered early in its course based on lack of CTC response. Results from this trial may allow refinement of response evaluation and, in particular, prevent unnecessarily prolonged treatment with an ineffective agent. 

Rather than using CTC count alone, assessment of the molecular characteristics of CTCs might be useful. Theoretically this could enable improved treatment selection and better prediction of response to a specific therapy. Molecular analysis studies of CTCs have previously been hampered by limited ability to detect and enrich for CTCs from a high background of lymphocytes. However, recent advances in isolation and enrichment techniques now allow more detailed assessment of CTCs, including analysis of molecular characteristics or expression of biomarkers, with potential treatment implications.

## 2. CTCs for Predictive Biomarker Assessment

### 2.1. Alteration in Receptor Status in MBC

The estrogen receptor (ER), progesterone receptor (PgR) and HER2 receptor are the only three validated biomarkers routinely applied in breast cancer management. ER, PgR and HER2 expression provide prognostic information, and inform on decisions regarding therapy. Endocrine therapy is only effective in the setting of ER and/or PgR positive disease, while anti-HER2 therapies are effective against those tumors overexpressing HER2. Currently, treatment decisions at the time of MBC relapse are generally made based on the receptor status of the primary breast cancer. However, there has been increased awareness of the possibility of discordance in receptor status between primary tumor and disease recurrence, leading to suggestions that reassessment of receptor status at the time of disease recurrence should be recommended. 

One of the hallmarks of cancer is genomic instability [17], indicating that genetic mutations, and hence alterations in both downstream pathway signalling and protein expression, are possible, if not expected. Thus, there exists the potential for alterations in tumor characteristics, in particular predictive biomarkers such as ER, PgR and HER2. 

Discordance between primary tumor and MBC tissue biopsies occur at rates of around 10%–30% for ER and 20%–50% for PgR, with losses being more common than gains. HER2 discordance has been reported in around 10% of paired primary and metastatic tumors, with loss and gain occurring with relatively equal frequency [18]. While there is reasonable rationale for the development of receptor discordance, the vast majority of data have been derived retrospectively, creating some uncertainty about the “true” discordance rate that may exist between primary and recurrent breast cancer. Of note, gain or loss of receptor status in metastatic disease based on tissue biopsies can alter treatment decisions [19,20], although, as yet, there is minimal evidence that alteration of treatment due to discordance leads to significant improvements in clinical outcomes [18]. 

### 2.2. CTCs: “Liquid Biopsies”

Interventional radiology and imaging now allow the option of obtaining a biopsy from the vast majority of metastatic sites. While tissue biopsies are relatively cheap, well validated, and generally easy to obtain, they are not benign procedures and can cause pain, minor bleeding or infrequently, more severe complications such as pneumothorax or haemorrhage. Analysis of receptor status on CTC is therefore an attractive option, as it might allow “real-time” analysis of ER, PR, HER2 plus other clinically relevant biomarkers to direct therapy from a simple blood test, that is, a “liquid biopsy” (see Table 1). Furthermore, repeated blood tests are much more feasible than repeated tissue biopsies, making serial assessment of biomarker status a realistic option for monitoring treatment response or alterations in tumor biology with therapy. Critically, the potential advantages of “liquid biopsy” over that of tissue biopsy are currently outweighed by barriers including cost, limited access to analytical equipment and most importantly, lack of validation of biomarkers on CTCs as useful in helping to dictate management. Ongoing development of CTC analysis may overcome some of these disadvantages, however at present, tissue biopsy remains the gold standard for evaluating suspected recurrent breast cancer.

**Table 1:** CTCs compared with tissue biopsy for biomarker assessment in metastatic breast cancer.

Tissue biopsy	CTC analysis: “Liquid biopsy”
Invasive, can infrequently cause significant morbidity	Minimally invasive
Monitoring treatment response/disease course with multiple biopsies generally not feasible	Monitoring treatment response/disease course with multiple samples relatively easily achieved
High likelihood of obtaining adequate tissue for analysis	CTCs can be hard to isolate or may be missed
Relatively cheap	Expensive
No specialised analytical equipment required	Specialised analytical equipment required
Can be performed at the vast majority of treatment centres	Can only be performed in certain laboratories equipped for CTC analysis
Interpretation of IHC (+/− FISH) assessment of tumor tissue standardised for ER, PgR, HER2	Further validation of best method for interpretation of HER2 or ER expression on CTCs needed
Clinical impact of treatment decisions based on tissue biopsy biomarker assessment uncertain	Clinical impact of treatment decisions based on CTC biomarker assessment uncertain

### 2.3. Biomarker Assessment Using CTCs

#### 2.3.1. CTC Isolation

A major challenge in the investigation of CTCs is that they are rare, and thus their detection is particularly challenging. The ratio of CTC to lymphocytes is in the order of 1 to 1,000,000, while it is around one in a billion for CTCs compared with erythrocytes. Given their rarity, the initial step in CTC analysis is their isolation, or enrichment, from other normal blood cell components. There are numerous technologies in development for this step, with the most widely used being CellSearch^®^ (Janssen Diagnostics, LLC Oncology Diagnotics, Raritan, NJ, USA) [21,22]. CellSearch^®^ is a semiautomated method that isolates CTCs by firstly applying an antibody for the epithelial cell adhesion molecule, EpCAM, which is often present on carcinoma cells including breast cancer, but not present on normal blood components. EpCAM antibodies are attached to microscopic iron particles, thus when a magnetic field is applied across the blood sample, EpCAM positive cells are isolated. CTCs are then identified by expression of cytokeratin 8, 18, 19 and lack of expression of CD45, the latter being a lymphocyte marker. Finally the isolated cells are assessed morphologically for large nuclei and size as well as other characteristics of malignancy [21]. 

This principle of an antibody (most frequently EpCAM) conjugated to magnetic particles is an immunomagnetic method, and forms the basis of CTC enrichment in other technologies including AdnaTest^®^ (AdnaGen AG, Langerhagen, Germany), MACS (Magnetic Activated Cell Sorting system) [23], and MagSweeper [24]. For instance, AdnaTest BreastCancerSelect/Detect™ isolates CTCs with use of EpCAM and MUC1 antibodies conjugated to magnetic beads, with a magnetic particle concentrator used to extract the labelled cells [25].

Importantly, there is increasing evidence that CTCs are a heterogeneous population of cells and that the most invasive and aggressive phenotype is associated with an epithelial to mesenchymal transition (EMT) and stem cell-like portrait. In this context, an enrichment technique that relies on EpCAM might be ineffective at selecting cells undergoing EMT progression, whereby EpCAM expression is lost. Consequently, some researchers are now evaluating the use of EMT markers to select CTCs [26,27].

Other enrichment methods separate CTCs from normal blood components based on physical properties, for example differing density-gradients (RosetteSep™ [28], OncoQuick^®^ (Greiner Bio One, Munich, Germany) [29]), or differing size (ScreenCell^®^ [30]). Density gradient techniques permit the separation of mononuclear and tumor cells, based on their lower density, from other blood elements. Alternatively, microfluidic chip technology (CTC-Chip [31], Herringbone (HB) Chip [32]) can allow CTC isolation. The CTC-Chip is a silicon microchip with thousands of microposts coated with anti-EpCAM. Whole blood is pushed over the chip surface, with EpCAM positive cells captured and stained with anti-cytokeratin antibodies before being analysed by fluorescence microscopy [31]. A potential improvement on the CTC-Chip is the HB chip, which follows similar principles of having CTCs captured with EpCAM antibodies, but instead of using microposts, the HB Chip design uses microvortices to cause a greatly enhanced number of collisions between CTCs and the antibody-coated chip surface, thereby improving CTC yield [32].

Few studies have directly compared enrichment methods. Müller *et al*. [33] demonstrated improved CTC yield with CellSearch^®^ compared with AdnaTest^®^ in a prospective trial of MBC patients, while Punnoose *et al*. found that CellSearch^®^ and CTC-Chip performed similarly as detection methods for CTCs in cell lines and whole blood samples from healthy donors, breast, and lung cancer patients [34]. Development and validation of numerous other enrichment methods are ongoing [35], and while results for some are promising, CellSearch^®^ is, at present, the only method adequately validated to receive FDA approval. 

#### 2.3.2. Molecular Analysis of CTCs

**Figure 1:**
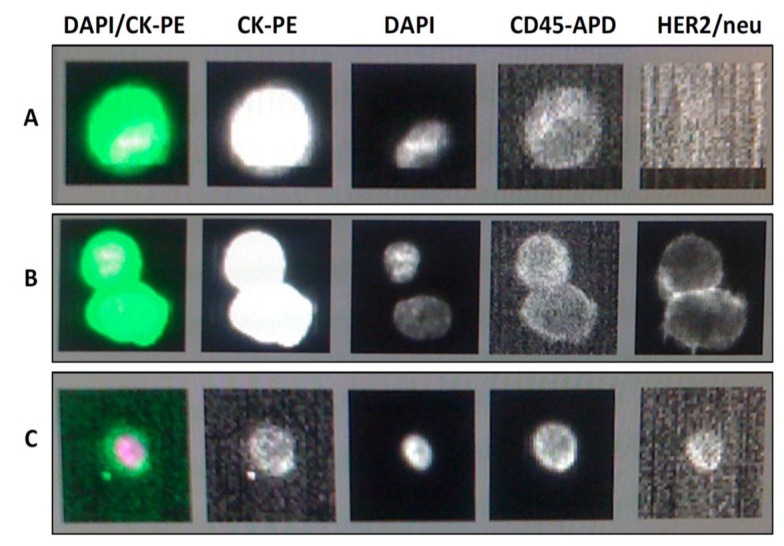
Typical appearance of isolated cells using the CellSearch^®^ method. DAPI: fluorescently stains nuclear material; CK-PE: detects presence of cytokeratin 8, 18, 19; CD45-APD: detects presence of CD45. (**a**)* HER2 negative CTC*: this cell is a CTC evidenced by CK positivity (2nd image from left) and DAPI positivity (3rd image) and CD45 negativity (4th image). HER2 immun(ofluorescence is negative (5th image); (**b**) *HER2 positive CTC:* CK is again positive, DAPI positive and CD45 negative consistent with this being a CTC. HER2 immunofluorescence is positive (5th image from left); (**c**) *Lymphocyte*: This cell is not a CTC as it is negative for CK (2nd image from left, compare with the two images above). DAPI positivity and CD45 positivity indicates this is a lymphocyte.

Just as there are various methods for isolating CTC from whole blood, assessment of biomarkers on CTCs can be achieved using different techniques: through evaluation of protein expression, mRNA expression, or chromosomal abnormalities. To evaluate protein expression on CTCs, the most well validated assay is the immunofluorescence (IF) staining for HER2 on single CTCs through the fourth filter on the CellTracks^®^ Analyzer digital microscope of the CellSearch^®^ System [36,37,38,39] (see Figure 1). During CTC enrichment in the CellTracks system cells are stained with fluorescein-conjugated anti-HER2/neu antibody, fluorescein-conjugated anti-EGFR antibody, or with phycoerythrin-conjugated anti-IGF-1R antibody, with the IF image then evaluable visually on a computer screen. This same procedure also allows cells to be stained for other proteins such as the neoepitope M30, which can identify cells in early phases of apoptosis after lab development [40]. 

Alternatively, after enrichment isolated CTCs can be transferred onto a coated slide and stained with an antibody of interest, or analysed using fluorescence *in situ* hybridization (FISH) to assess for chromosomal abnormalities [35,38,41]. FISH is predominantly applied to evaluate HER2 overexpression due to amplification of the HER2 oncogene located on the long arm of chromosome 17 (17q12) [42,43]. The American Society of Clinical Oncology (ASCO) and the College of American Pathologists (CAP) have developed guidelines for HER2 amplification in breast cancer: an absolute HER2 gene copy number lower than four or HER2/CEP17 ratio of <1.8 is considered HER2 negative (HER2^–^), an absolute HER2 gene copy number between four and six or HER2/CEP17 ratio between 1.8 and 2.2 is considered HER2 equivocal, and an absolute HER2 gene copy number greater than six or HER2/CEP17 ratio >2.2 is considered HER2 positive (HER2^+^) [44]. Importantly, while this same definition of HER2 positivity FISH analysis can be applied to single CTCs [38], the number and proportion of CTCs with elevated HER2/CEP17 ratio from the overall CTC population that constitutes HER2 positive disease is uncertain [41]. In addition, further evaluation of the role of polysomy 17 in CTCs is needed. Hayashi *et al*. recently reported that, from a small case series, seven of 49 MBC patients had polysomy 17 detected on CTCs, although no CTCs with polysomy 17 were also HER2 amplified on FISH analysis [45]. 

Regarding the expression of mRNA on CTCs, this is assessed using real-time reverse transcription polymerase chain reaction (RT-PCR) [35,46]. Following collection of whole blood samples in EDTA, CTCs are selected using the CellSearch^®^ system, and after a process of nucleic acid enrichment, mRNA expression can be evaluated quantitatively [47]. Moreover, using the commercially available AdnaTest^®^, after CTC enrichment and lysis, mRNA is isolated and retrotranscribed in cDNA, cDNA can be tested for ERBB2 (HER2) mRNA [48], “stemness” markers, such as ALDH1, or markers of epithelial-mesenchymal transition (EMT) including Twist1 and Akt2 [49]. As the CTC is lysed in this process, morphological assessment of the CTC or quantification of number of CTCs expressing the specified biomarker are not possible.

Technologies other than AdnaTest^®^ are being developed that allow mRNA assessment. For instance, after HB Chip CTC enrichment, Yu *et al*. was able to evaluate EMT markers on these CTCs using QuantiGene View RNA *in situ* hybridization (Affymetrix, Santa Clara, CA, USA). Furthermore, they demonstrated that the presence of CTCs with EMT characteristics correlated with disease progression [50].

A further analysis technique worth mentioning is that of DEPArray^®^ (Di-Electro-Phoretic Array system; Silicon Biosystems, Bologna, Italy), a novel semiautomated system comprising of a chip with microelectrode that create electric cages in which single CTCs are effectively trapped [51]. Ideally, samples should be enriched prior to use of DEPArray™, which can be achieved using CellSearch^®^. Following isolation of single pure CTCs, the cells’ DNA is amplified and sequenced, and genetic markers of interest assessed. As the CTCs collected are pure, downstream analyses are much less likely to be confounded with contaminants. 

In a previous study from our group that compared techniques for determining HER2 status on CTCs, good concordance between IF and FISH was demonstrated. From 25 evaluable MBC patients with both FISH and IF analysis of CTCs, 19 of 20 patients with HER2 negative CTCs on FISH were also IF negative, while four of five patients with HER2 amplification had concordant IF results [38]. This correlation between techniques is encouraging, however these data are preliminary. Further prospective validation of the various methods for HER2 evaluation on CTCs is required before they can be reliably applied in clinical practice. 

## 3. CTC Biomarker Analyses in the Management of MBC

The majority of studies assessing biomarker expression on CTCs have been focused on HER2, with limited data on hormone receptors or other putative biomarkers. The HER2 status has therapeutic implications, being a strong predictive marker for clinical benefit from HER2-targeted therapies such as trastuzumab and lapatinib, in both the metastatic and adjuvant breast cancer settings [52,53,54,55,56]. HER2 overexpression on CTCs might theoretically therefore provide patients with an additional treatment option, specifically in the setting of a HER2 negative/unknown primary tumor.

### 3.1. HER2 Data

Of the studies that have assessed HER2 expression in CTCs, the majority have evaluated discordance rates between CTC HER2 expression and that of the primary breast cancer, with few assessing the prognostic role of HER2^+^ CTCs or the efficacy of anti-HER2 therapy.

#### 3.1.1. HER2^+^ CTCs and Prognosis

HER2 positivity is a known poor prognostic factor [57,58,59] with HER2^+^ breast cancers typified by aggressive clinical course and early relapse. Much less is known about the prognostic implications of HER2^+^ CTCs. In a study of 76 patients with MBC from Munzone *et al*. [60], those with HER2^+^ CTCs at baseline after HER2- primary breast cancer had significantly shorter PFS than those who retained HER2^−^ status on CTCs, and patients with no CTCs (15 weeks *vs*. 20 weeks *vs*. 25 weeks, for HER2^+^ CTCs, HER2^−^ CTCs, and no CTCs, respectively) [60]. Similarly, in a small study from Hayashi *et al*. [36], PFS and OS were both significantly shorter in six patients with MBC and HER2^+^ CTCs, compared with 43 MBC patients without HER2^+^ CTCs. In a multivariate analysis, the presence of HER2^+^ CTCs was an independent prognostic factor along with the number of prior therapies, while CTC count, although not HER2^+^ CTCs specifically, was an independent prognostic factor for OS [36]. Identification of HER2^+^ CTCs, as defined by expression of HER2 mRNA derived from CTCs, was also associated with shorter disease free survival (DFS) in a cohort of early breast cancer patients, although this did not remain an independent prognostic factor in a multivariate analysis, nor was there any significant association with OS [61]. Similarly, in a cohort of 35 stage I-III breast cancer patients, the presence of HER2^+^ CTCs was associated with worse DFS and OS. In a multivariate analysis HER2^+^ CTCs and T stage were the only independent prognostic factors, although importantly, CTC count per se was not included as a covariate [62]. 

An additional confounding factor regarding the prognostic role of HER2^+^ CTCs is the potential influence of anti-HER2 therapy. Giordano *et al*. recently reported that, in a cohort of over 500 MBC patients, CTC count retained prognostic significance in ER^+^ and triple negative subtypes, but this was lost in patients with HER2^+^ disease after trastuzumab treatment [63]. Munzone *et al*. similarly found that a CTC count of 0 per 7.5 mL of whole blood was associated with better prognosis in all subtypes except HER2^+^, further suggesting this may have been due to the impact of targeted therapy, though no data on anti-HER2 therapy received was reported [64]. 

#### 3.1.2. HER2 Discordance

Discordance between primary breast cancer and HER2 expression on CTCs in the setting of disease recurrence has been reported at variable rates, with rate of gain of HER2 from 9% to over 60% (see Table 2). Importantly, as well as the varying methods of CTC enrichment and HER2 detection utilised, the definition of HER2 positivity is widely ranging across studies. For instance, Ignatiadis *et al*. defined HER2 positivity as the presence of ≥1 CTC expressing HER2 with IF staining intensity of at least 2.5 times background [37]. In contrast, Ligthart *et al*. used threshold of at least five CTCs detected, of which ≥75% had to be HER2 positive [65]. In a prospective study from Fehm *et al*. [48], whether a patient was considered to be “HER2 positive” after CTC analysis was dependent on the definition employed. Using a ratio of HER2^+^ CTCs to total CTCs of >10% compared with HER2^+^ to total CTC ratio of >50% resulted in the proportion of patients being classed “HER2 positive” of 64% and 26%, respectively [48]. It is worth noting also that AdnaTest^®^ or other RT-PCR based techniques do not allow quantification of the number of HER2^+^ CTCs, hence any HER2 mRNA detected could be considered positive. 

**Table 2:** Studies assessing HER2 discordance between primary tumor and HER2 on CTCs in recurrent breast cancer. ^ Includes only patients with metastatic breast cancer; * includes only patients with known HER2 primary tumor status. FISH: fluorescence *in situ* hybridization; IF: immunofluorescence; Immunomag: immunomagnetic technique; RT-PCR: real-time reverse transcription polymerase chain reaction.

Author [ref.]	Year	No. of pts ^	No. (%) with CTCs	CTC analysis	HER2 assessment	Rate of discordance*	
						HER2^+^→HER2^−^	HER2^−^→HER2^+^
de Albuquerque [66]	2012	32	24 (75%)	Immunomag	RT-PCR	8/9 (89%)	4/15 (27%)
Fehm [67]	2007	77	21 (27%)	Immunomag	IF and FISH, some with RT-PCR	2/3 (67%)	4/12 (33%)
Fehm [48]	2010	254	122 (48%)	CellSearch^®^	IF	13/31 (42%)	25/76 (33%)
		229	90 (39%)	AdnaTest^®^	RT-PCR	13/22 (59%)	28/57 (49%)
Flores [68]	2010	75	75 (100%)	CellSearch^®^	FISH	1/45 (2%)	10/30 (33%)
Ignatiadis [37]	2011	39	23 (59%)	CellSearch^®^	IF	1/2 (50%)	13/21 (61%)
Ligthart [65]	2013	103	90 (87%)	CellSearch^®^	IF (automated)	29%	9%
Meng [41]	2004	24	24 (100%)	Immunomag	FISH	-	9/24 (38%)
Munzone [60]	2010	76	57 (75%)	CellSearch^®^	IF	2/15 (13%)	6/42 (14%)
Pestrin [38]	2009	66	40 (61%)	CellSearch^®^	IF	5/12 (42%)	8/28 (29%)
Punnoose [34]	2010	38	29 (76%)	CellSearch^®^	IF	3/12 (25%)	2/17 (12%)
Somlo [69]	2011	22	18 (81%)	MACS	IF	3/5 (60%)	3/13 (23%)
Tewes [14]	2009	42	22 (52%)	AdnaTest	RT-PCR	3/5 (60%)	5/17 (29%)

#### 3.1.3. Treatment with Anti-HER2 Therapy for HER2^+^ CTCs

Arguably the most important factor relating to HER2 analysis on CTCs is whether targeted treatment of HER2^+^ CTCs in a patient with HER2^−^ disease is effective. As yet, there are minimal data addressing this. Meng *et al.* [41] reported in their retrospective study of 24 patients with MBC and HER2^−^ primary tumor, that four of nine patients with HER2^+^ CTCs at the time of metastatic disease received trastuzumab. Of these, one had rapid remission of symptoms and complete response on imaging, two patients had partial responses and one no response [41].

Conversely, minimal activity from anti-HER2 therapy was evident in an open label phase II trial from Pestrin *et al*. In this prospective feasibility study, patients with recurrent disease after previous HER2^−^ primary breast cancer were screened for the presence of CTCs. From 139 screened patients, 96 had at least five detectable CTCs per 7.5 mL whole blood, and seven (7%) were HER2 positive, with HER2 positivity defined as ≥50% of CTCs staining HER2^+^ on CellSearch^®^ IF assay. These seven patients were commenced on lapatinib therapy, 1500 mg/day. There were no observed responses, while one patient has stable disease lasting 8.5 months [70]. Of note, recruitment of this study was hampered by the low rate of HER2 positivity on CTCs as defined by the eligibility criteria. 

In a parallel trial from Stebbing *et al*. [71], the feasibility of lapatinib treatment in MBC patients with EGFR-expressing CTCs was assessed. Patients were required to have an EGFR negative primary tumor and EGFR positivity on CTCs. In this case, the threshold for positivity was ≥ 1 CTC expressing EGFR. From 43 screened patients, 16 were eligible, with two withdrawing for toxicity to lapatinib after commencement of treatment. There were no objective responses or stabilization of disease, with all 16 patients progressing within 12 weeks of treatment [71]. While depletion of the EGFR^+^ CTC pool was observed in four patients on treatment, this did not appear to result in a meaningful clinical effect. The small number of included patients limits interpretation of the results from both these trials, highlighting the need for additional prospective studies.

Interestingly, Liu *et al*. also reported reduction in the pool of EGFR^+^ CTCs with lapatinib treatment in a case report of a woman with chemotherapy-refractory HER2^+^ MBC. Decreased EGFR^+^ CTC count coincided with treatment response, while at the time of disease progression, increased EGFR negative and HER2 negative CTCs were evident [72]. 

#### 3.1.4. Ongoing Studies of Relevance

Based on the results from previous studies demonstrating alteration, in particular gain, in HER2 status during progression to MBC [48,67], the DETECT III randomised phase III trial (*NCT01619111*) was launched by the same group in early 2012. This multicentre study compares standard therapy with or without lapatinib in patients with MBC after HER2^−^ primary tumor but with HER2^+^ CTCs [73] (see Table 3), with the underlying concept being that acquisition of HER2 positivity on CTCs may present patients with the alternate treatment option of anti-HER2 therapy. While the clinical implications of HER2 discordance in MBC are uncertain, the rationale for a trial evaluating an approach that could improve treatment response seems reasonable. Should the DETECT III trial demonstrate an advantage for the addition of anti-HER2 therapy in the setting of apparent HER2 gain, treatment for patients might also potentially expand to include other anti-HER2 agents. Of note, this trial was planned prior to results from the prospective phase II trial from Pestrin *et al*. being published. The results of this larger trial will be of interest, particularly in light of the negative results from the phase II trial. A further trial in development evaluating anti-HER2 therapy efficacy in the setting of HER2^+^ CTCs is the provisionally named CirCè XXX1 trial [74].

One additional trial worth mentioning is the EORTC sponsored trastuzumab in HER2-negative Early Breast Cancer as Adjuvant Treatment for Circulating Tumor Cells (TREAT CTC, *NCT01548677*). The trial aims to determine if, using trastuzumab to facilitate antibody dependent cell-mediated cytotoxicity (ADCC), HER2^+^ CTCs can be eradicated, leading to improved clinical outcomes (see Table 3). Of note, while this is an adjuvant therapy trial, with ADCC potentially considered more relevant in the adjuvant than the advanced breast cancer setting, the rationale represents an alternate approach to CTC (in this case micrometastatic disease) eradication and results are anticipated with interest.

### 3.2. Other Biomarkers

#### 3.2.1. ER/PgR

Data on other established or putative biomarkers are sparse. Aktas *et al*. [75] reported that, in a cohort of 193 prospectively evaluated MBC patients, of whom 45% had CTCs detectable, less than half showed concordance between ER and PgR expression. Predominantly, discordance was due to loss of hormone receptor expression, occurring in 77% and 87% of ER^+^ and PgR^+^ tumors, respectively [75]. This is consistent with data on ER and PgR expression of primary tumors compared with metastatic tissue biopsy, where receptor loss is much more common than gain [18]. However, the clinical implications of hormone receptor loss on CTCs are unknown. Trials of endocrine therapy in the setting of apparent loss would generally be recommended, due to the relatively low toxicity rate from endocrine therapies and the potential for a false negative result. To further assess the predictive role of hormone receptor expression on CTCs prospective evaluation is needed, similar to that currently being undertaken for HER2.

**Table 3:** Summary of DETECT III and TREAT CTC trial characteristics. CBR: clinical benefit rate; FISH: fluorescence *in situ* hybridisation; (I)DFS: (invasive) disease free survival; IHC: immunohistochemistry; MBC: metastatic breast cancer; NA: not applicable; ORR: objective response rate; OS: overall survival; PFS: progression free survival; QoL: quality of life; RFS: relapse free survival.

Trial type	DETECT III	TREAT CTC
	Phase III randomised controlled trial	Phase II randomised controlled trial
Aim	To compare standard therapy alone *versus* standard therapy plus lapatinib in patients with initially HER2-negative MBC and HER2-positive CTC	To compare trastuzumab *versus* observation in patients with HER2 negative early breast cancer and detectable HER2-positive CTC after (neo)adjuvant therapy and surgery
Rationale	HER2^+^ CTCs may indicate presence of HER2^+^ metastatic disease and increased downstream proliferation due to HER2 activation. This pathway activation might be blocked by anti-HER2 therapy	Trastuzumab may facilitate anti-cancer activity through activation of antibody dependent cell-mediated cytotoxicity against HER2^+^ CTCs, rather than direct HER2 inhibition
Control arm	Standard therapy options: aromatase inhibitors, taxanes, capecitabine, vinorelbine, non pegylated liposomal doxorubicin	Observation
No. of previous chemotherapy lines for MBC permitted	≤3	NA
Primary tumor	HER2 negative	HER2 negative (mandatory central confirmation)
Requirement for CTC	≥1 HER2^+^ CTC as determined by IHC or FISH, per 7.5 mL whole blood	≥1 HER2^+^ CTC per 15 mL whole blood
Primary outcome measure	PFS	CTC detection at week 18
Secondary outcome measures	ORR; CBR; OS; QoL; safety, pain intensity, CTC count dynamics	RFS; IDFS; DFS; OS; safety; CTC assay and correlation
Estimated enrolment	228	2,175
Estimated primary completion date	March 2016	January 2015

#### 3.2.2. *PIK3CA* Mutations

Activation of the PI3K/Akt/mTOR signalling pathway can lead to increased cell proliferation and dysfunction of tumor suppression function [76]. Mutations or loss of expression in components of this pathway occur frequently in many cancer types, particularly luminal or ER^+^ breast cancers. The most commonly reported mutation is that of phosphatidylinositol-4,5-bisphosphate 3-kinase, catalytic subunit α (*PIK3CA*), although the clinical implications of mutations in this gene are unclear. While dysfunction in PI3K pathway can increase tumorogenesis, retrospective data suggest that outcomes might be more favourable in patients with ER^+^ breast cancer expressing a *PIK3CA* mutation [77]. 

Despite the uncertainty regarding the prognostic impact of *PIK3CA* mutations, this gene is of significant interest given both the prevalence of reported mutations, and the existence of a number of targeted therapies already in clinical development against this pathway. Thus, evaluation of *PIK3CA* mutations on CTCs might have clinical relevance. Following enrichment of blood samples from 44 MBC patients with CellSearch^®^, Schenk *et al*. were able to isolate, amplify and analyse genomic DNA for *PIK3CA* mutations in the known mutations hotspots of exon 9 and 22. Mutations were detected in 7 out of 44 patients (16%), although the frequently reported E542K, E545G and E545A variants were not detected [78].

Our group has recently reported an alternative feasible method for the molecular assessment of CTCs, in particular *PIK3CA* mutations, in MBC. Utilization of CellSearch^®^ followed by DEPArray™ enabled recovery of single pure CTCs in patients with MBC. Using whole genome amplification (WGA) and sequencing reactions on single CTC DNA samples, mutations in the *PIK3CA* gene were identified in 4 out of 13 evaluable patients [79]. In an exploratory analysis, six cases with more than one CTC sequenced had no heterogeneity in mutational status of *PIK3CA* gene. While significantly limited in numbers at present, this pilot study demonstrates that isolation and molecular analysis for *PIK3CA* mutation on single CTCs in MBC patients is feasible. Ongoing planned research includes further validation of this method and evaluation of *PIK3CA* mutational status defined on CTC compared with that of tumor tissue from primary or metastatic disease. While this approach demonstrated feasibility, ultimately prospective evaluation of targeted agents against aberrant PI3K signalling pathway in patients with *PIK3CA* mutation positive CTCs would be required to clarify the clinical utility of this approach. 

#### 3.2.3. Chemotherapy Biomarkers

Chemotherapy is effective in the treatment of MBC, however, not all those patients treated will respond, although all will be subjected to the toxic side effects. Results from neoadjuvant chemotherapy trials suggest tumor biology is an important factor in determining response to chemotherapy, whereby luminal A breast cancer is relatively chemoresistant, with other subtypes are chemosensitive [80]. Otherwise, the decision to commence chemotherapy is typically made based on patient factors, extent and clinical trajectory of disease, and alternate treatment options. Response to treatment is evaluated using clinical and radiological means, with neither of these approaches consistently allowing early detection of treatment resistance that might enable early cessation of an ineffective therapy. 

There are preliminary data suggesting that CTCs might be useful in chemotherapy response prediction both at baseline and after treatment commencement. CTC count has been suggested as a marker of chemotherapy response in MBC [14,15]. Furthermore, in a study of 34 newly-diagnosed MBC patients, Cheng *et al*. reported that detection of MUC-1 mRNA from CTCs after the first cycle of chemotherapy was significantly associated with poorer response rate compared with patients with undetectable MUC-1 mRNA, while there was a trend towards increased PFS in MUC-1 mRNA negative patients. MUC-1 mRNA prior to commencement of chemotherapy showed no correlation with efficacy of chemotherapy [81].

An extension of the concept of evaluation of chemosensitivity in general is that of evaluation of sensitivity to specific chemotherapeutic agents, of which there are currently no known validated biomarkers. Conflicting and inconclusive data exist for the putative biomarker TOP2A and response to anthracyclines [82,83,84], while mutant p53 as a marker of taxane sensitivity has been shown to be of little clinical use [85,86]. Using molecular analysis of CTCs, Gazzaniga *et al*. [87] investigated the ability of a panel of genes for multi drug resistance proteins (MRPs) associated with resistance to specific chemotherapeutic agents to differentiate chemotherapy responders from non-responders. From 105 cancer patients with either early stage or advanced disease, a minority (n = 14) of whom had breast cancer, CTCs were detected in 54 patients (51%). Patients were classified as chemotherapy “resistant” or “sensitive” based on expression of MRPs on CTCs correlated with the specific chemotherapeutic regimen they received. From 32 advanced cancer patients, 21 developed progressive disease, 20 of whom had a “resistant” profile. Similarly, for the 11 patients with disease stabilization, there was 100% correlation with a “sensitive” profile. Furthermore, in a univariate analysis, there was a statistically significant correlation between the CTC drug-resistance gene profile and both time to progression in metastatic disease and disease free survival in early stage disease [87]. 

These data are very preliminary and limited by low patient numbers, yet intriguing, and further research in this area would be of particular interest to improve efficacy and reduce unnecessary toxicities from chemotherapy in treatment of MBC. 

## 4. Issues and Challenges

### 4.1. Technical Challenges with CTC Analysis

#### 4.1.1. Detection of CTCs

As CellSearch^®^, along with several other CTC enrichment techniques, relies on the presence of epithelial cell markers, CTCs that do not express EpCAM, such as those that have undergone EMT may be missed [88,89]. EMT is a process thought to be critical in the ability of a cancer to metastasise. Transition to a mesenchymal phenotype, and thus loss of expression of the epithelial marker, EpCAM, allows cell migration, while re-establishment of epithelial phenotype at the site of metastasis promotes cell proliferation, growth and establishment of a new site of disease. Detection of higher rates of CTCs in ER^+^ compared with higher risk tumors has been reported [34,64]. Although higher risk tumors might be expected to have higher CTC counts given their higher propensity to recur, a counterintuitive decrease in CTC detection could relate to EMT, which has been reported to occur at increased rates in certain high-risk tumor types, such as basal-like and triple negative breast cancer [90,91,92].

There is a need to improve CTC detection techniques, in particular focusing on methods for identifying CTCs even in the setting of EMT. One such approach might be through use of antibodies against Twist1, Akt2 or other EMT markers [49]. For instance, Bitting *et al*. recently reported development of a mesenchymal-based method of isolating CTCs in men with metastatic prostate cancer based on surface expression of OB-cadherin [93]. The utility of this method in breast cancer has yet to be evaluated. Furthermore, there is evidence that “normal-like” breast cancer cell lines do not express EpCAM, in comparison with other intrinsic subtypes, and hence can be missed using this antibody alone [94]. CD146 is instead frequently expressed, and the addition of anti-CD146 to EpCAM was recently demonstrated to improve the recovery of CTCs in a cohort of 20 MBC patients [26]. Finally, in addition to improving the ability to detect CTCs undergoing EMT, or other CTCs not expressing EpCAM, it is important to be able to identify viable cells from those that have undergone apoptosis, something that might be achieved using an antibody against M30 neoepitope [40]. 

#### 4.1.2. What Is the Best Technique for Molecular Analysis?

While there is only one FDA approved method for CTC enrichment, many others are in development and show promising preliminary results. Studies comparing different technologies might be useful to suggest the relative sensitivity and specificity of each. In order for CTC molecular analysis to enter routine clinical practice in the future, enrichment of CTCs and the subsequent method of CTC analysis should ideally be standardised, so that results obtained might be applied more easily across patient populations.

In particular, differences in rates of HER2 positivity (or other molecular markers) can be seen depending on which detection and analytical method is applied [48]. This makes correlation between molecular marker expression and clinical response to a targeted therapy against that marker critical in order to permit application of CTC molecular analysis in a clinical context.

#### 4.1.3. Standardising Definitions of Biomarker Expression

Since formulation of the ASCO and CAP guidelines for defining HER2 positivity, there is reasonable reliability of HER2 results obtained from tissue samples. Molecular analysis on CTCs is a relatively new field and as yet, it is not known how best to define a patient as having HER2^+^ disease based on CTC HER2 status. While the assays to detect HER2 on CTCs are themselves reliable, interpretation of these results and their utility in a clinical context are not. Given the known heterogeneity of HER2 overexpression within single tumors, not all CTCs would be expected to be HER2^+^, but the optimal cut-off for the proportion of HER2^+^ CTCs to define a patients CTC-HER2 positive is yet to be determined. Standardisation of the definition of HER2 positivity can only be achieved through evaluation in a prospective clinical trial setting. Employing a definition of HER2 positivity that results in no response to anti-HER2 therapy has little clinical relevance. It might be possible to evaluate this issue within clinical trials of anti-HER2 therapies by selecting a lower threshold for HER2 positivity, with evaluation of response in subgroups based on number and proportion of HER2^+^ CTCs.

#### 4.1.4. Feasibility

CTC analysis remains a relatively costly research tool at present, primarily due to the cost of the analytical equipment. In addition, expertise is required to perform CTC analysis, limiting access of this technology to dedicated research laboratories. Ongoing expansion of this field however, will ideally see a decrease in the costs related to access to CTC technologies, as well as an increase in the number of skilled researchers with expertise in interpretation of CTC molecular analyses.

### 4.2. Limited Patient Numbers

Studies evaluating CTC molecular characterisation are all relatively limited in terms of number of included patients. As such, results across all studies should be interpreted with caution and no firm conclusions be drawn. Unlike the prognostic utility of CTCs in MBC, which has been well established and validated in prospective trials, predictive biomarkers on CTCs lack adequate prospective validation and therefore are not yet ready to be applied in a clinical setting.

### 4.3. Heterogeneity

CTCs are rare, and analysis of biomarker expression, such as HER2, on CTCs provides markedly less comprehensive assessment of the entire tumor than might a tissue biopsy, where hundreds of cells can be analysed. There is minimal data available on heterogeneity of biomarkers on CTCs, raising the question of whether molecular analysis results are fully representative of the metastatic tumor. Furthermore, tumor heterogeneity in tissue samples is well recognised, increasing the probability that CTCs expressing differing molecular profiles might be seeded into the bloodstream. Research into the impact of heterogeneity is crucial, and specifically if, in the setting of discordance with the primary tumor, treatment should be targeted against the most aggressive characteristic found, or, alternatively, the most common. Indeed, it will not be possible to reliably interpret CTC biomarker results in a clinical context until our understanding of CTC biomarker heterogeneity improves.

### 4.4. Clinical Relevance of Biomarkers and Interpretation of CTC Biomarker Results

Biomarker analysis of single CTCs is now becoming a feasible approach analytically, although, critically, the clinical relevance of biomarker expression on CTCs is unknown. In particular, there is no evidence that treatment of patients’ metastatic disease based on the CTC molecular profile, rather than the primary tumor, improves clinical outcomes. Studies that have assessed HER2 discordance between primary breast cancer and tissue biopsy of metastatic disease report alterations in treatment administered due to rebiopsy, with some suggesting treatment response to anti-HER2 therapy with HER2 gain, however these data are very limited [18]. In order for CTC analysis to be adopted as a means of directing treatment decisions in MBC, prospective studies addressing this clinical question are required. The results from the DETECT III trial are therefore awaited with considerable interest.

## 5. Conclusions

Molecular analyses on CTCs are feasible, although there are many questions that must be addressed in terms of reproducibility, assay sensitivity, reliability of results and clinical relevance before this approach can be considered suitable for routinely guiding management in MBC. Although the existing challenges that must be met in order to translate CTC biomarker analysis into a technique that can improve outcomes in MBC patients are substantial, the potential benefits are at least as, if not more, substantial. Such benefits include improved prognostication, increased understanding of breast cancer disease processes, feasible disease monitoring on therapy, and adjustment of treatment based on real-time biological changes in cancers. With ongoing development of this field, CTC analysis might provide us in the future with a relatively simple, repeatable and reproducible assay to help tailor treatment for individual MBC patients, leading ideally to improved outcomes in this disease. 
